# Prevalence and Factors Associated With Depression and Suicidal Ideation During the COVID-19 Pandemic Among University Students in Uganda: A Cross-Sectional Study

**DOI:** 10.3389/fpsyt.2022.842466

**Published:** 2022-04-14

**Authors:** Mark Mohan Kaggwa, Innocent Arinaitwe, Elicana Nduhuura, Moses Muwanguzi, Jonathan Kajjimu, Moses Kule, Noble Ajuna, Ivan Machacha, Rahel Nkola, Sarah Maria Najjuka, Nicholas Kisaakye Wamala, Felix Bongomin, Mark D. Griffiths, Godfrey Zari Rukundo, Mohammed A. Mamun

**Affiliations:** ^1^Department of Psychiatry, Faculty of Medicine, Mbarara University of Science and Technology, Mbarara, Uganda; ^2^African Centre for Suicide Prevention and Research, Mbarara, Uganda; ^3^Faculty of Medicine, Mbarara University of Science and Technology, Mbarara, Uganda; ^4^Department of Psychiatry, Mbarara Regional Referral Hospital, Mbarara, Uganda; ^5^Department of Nursing, Bishop Stuart University, Mbarara, Uganda; ^6^School of Medicine, Kabale University, Kabale, Uganda; ^7^College of Health Sciences, Makerere University, Kampala, Uganda; ^8^Faculty of Clinical Medicine and Dentistry, Kampala International University, Kampala, Uganda; ^9^Department of Medical Microbiology and Immunology, Faculty of Medicine, Gulu University, Gulu, Uganda; ^10^Department of Psychology, Nottingham Trent University, Nottingham, United Kingdom; ^11^CHINTA Research Bangladesh, Savar, Bangladesh; ^12^Department of Public Health and Informatics, Jahangirnagar University, Savar, Bangladesh; ^13^Department of Public Health, Daffodil International University, Dhaka, Bangladesh

**Keywords:** depression, university students, sexual abuse, academic satisfaction, COVID-19, suicidal ideation

## Abstract

**Background:**

The COVID-19 pandemic has negatively impacted psychosocial well-being and mental health of students across the world. Although students are vulnerable to depression and suicidal ideation, few studies have been conducted in Uganda. This study aimed to determine the prevalence of depression and suicidal ideation, and associated factors among undergraduate university students in Uganda.

**Methods:**

A cross-sectional study was conducted among undergraduates [*N* = 540; 363 males; mean age = 23.3 (± 2.64) years] recruited from four universities using an online questionnaire that explored sociodemographic factors, depression, and other associated factors. The Patient Health Questionnaire (PHQ-9) was used to assess depression, and Item 9 was used to assess suicidal ideation in the past 2 weeks. Multivariable logistic regression analyses were performed to determine the factors associated with depression and suicidal ideation.

**Results:**

The prevalence of moderate to severe depression was 20% (*n* = 108) (cut-off: 10/27 based on the PHQ-9), and the prevalence of past-2-week suicidal ideation was 13.89% (*n* = 75) (cut-off: 1/3 based on the PHQ-9 Item 9). About half of the individuals who screened positive for depression had suicidal ideation. Factors associated with depression were: having relationship issues [adjusted odds ratio (aOR) = 1.79, 95% confidence interval (CI) = 1.13–2.81, *p* = 0.012], and having a history of sexual abuse (aOR = 2.06, 95% CI = 1.10–3.84, *p* = 0.023). Factors associated with reducing the risk of depression were: satisfaction with current academic performance (aOR = 0.50, 95% CI = 0.32–0.79, *p* = 0.003), and being in the fifth year of academic study (aOR = 0.16, 95% CI = 0.03–0.73, *p* = 0.018). Factors associated with suicidal ideation were: smoking cigarettes and/or marijuana (aOR = 4.83, 95% CI = 1.10–21.12, *p* = 0.037), and having financial tuition constraints (aOR = 1.85, 95% CI = 1.08–3.16, *p* = 0.024), However, satisfaction with current academic performance reduced the likelihood of suicidal ideation (aOR = 0.40, 95% CI = 0.23–0.70, *p* = 0.001).

**Conclusion:**

Approximately one-fifth of undergraduate university students were moderately to severely depressed, especially those who had relationship issues and those with a history of sexual abuse. Suicidal ideation was common among smokers and those having financial tuition constraints. Therefore, it is recommended that the university authorities implement measures to provide psychological support for the students with problems concerning financial tuition constraints, relationships, and sexual abuse. Also, all students with depression should be screened for suicidality.

## Introduction

Depression is a mental health disorder characterized by extreme sadness, feelings of emptiness, and/or irritable mood, accompanied by somatic and cognitive changes that significantly affect the individual’s capacity to function (1). Globally, depression affects nearly 280 million people and can lead to a profound effect on all aspects of life, including a lower performance at school, poorer productivity at work, compromised relationships with family and friends, and lower ability to participate in the community (2). It was reported that Africa alone had 29.19 million cases of depression, accounting for 9% of the global depression burden (3). In Uganda, the general population prevalence was reported to be 4.6% (3). The prevalence of depression among university students in Uganda has ranged between 4.0 and 80.7% (4–7). The prevalence of depression in Uganda has been much higher among students during the coronavirus disease 2019 (COVID-19) pandemic (4) and a period characterized by marked psychological stressors and suicidality among students (8–12).

Various factors have been associated with the increased prevalence of depression among university students during the COVID-19 pandemic, including being female, precariousness, previous history of psychiatric illness, social isolation, COVID-19 illness, symptoms compatible with COVID-19, low quality of social relationships, low quality of COVID-19 information received, being a student aged 18–24 years, difficulties with paying for tuition fees prior to the pandemic, increased use of social media, internet use disorder, lack of physical activities/exercise, fear of COVID-19, poverty, and substance abuse (4, 13–16). In addition to COVID-19-related factors, several other factors have been associated with depression, including unhappy interpersonal relationships, chronic physical medical illnesses, chronic mental illnesses, low self-esteem, poor academic performance, family history of mental illness, financial constraints, insecurity at places of residence, smartphone addiction, being single, and the negative perception of the students of their learning environment (17–24). The effects of depression among students vary from mild effects such as poor academic performance (25) to very extreme events such as suicidal behaviors (9, 26–28). Previous studies have shown university students in Uganda to be at high risk of depression during the COVID-19 pandemic due to factors such as burnout, anxiety, and stress (4, 29), and such mood disorders also contribute to the increased risk of suicidal ideation (8).

Despite the introduction of peer support systems at the universities in Uganda, the prevalence of depression increased from 4.0% in 2002 to 21.5 in 2019 and 80.7% in 2020 during the first wave of the COVID-19 pandemic (4, 6, 7). This marked increase in the prevalence of depression over the years, especially during the COVID-19 pandemic, makes understanding depression among university students paramount, especially as the COVID-19 pandemic continues. Therefore, to have a better understanding of depression and suicidal ideation among students during the COVID-19 pandemic, a study among four universities in south-western Uganda was conducted during the second wave of the COVID-19 pandemic.

## Materials and Methods

### Study Design and Settings

This was a cross-sectional survey conducted among undergraduate university students between May 2021 and September 2021 in south-western Uganda. Participants were recruited using the convenience sampling method incorporating snowball sampling with the assistance of class coordinators and faculty representatives from the four universities: Bishop Stuart University (BSU), Kampala International University—western Uganda campus (KIU), Mbarara University of Science and Technology (MUST), and Kabale University (KU). BSU is located in Mbarara City (western Uganda) with approximately 5,800 students. KIU is located 60 km from Mbarara city with approximately 17,000 students. MUST is located in Mbarara City with approximately 4,260 students. KU is located in Kabale (in extreme south-western Uganda) with approximately 3,000 students.

### Inclusion and Exclusion Criteria

The study included students in KIU, BSU, KU, and MUST, aged 18 years or above during the academic year of 2019/2020, who agreed to take part in the study.

### Sample Size Calculation

Using Epi Info StatCalc for Population Surveys (Version 7.2.5.0), a population size of 30,000 undergraduate university students was used to calculate the minimum sample size required to produce statistical power of 80%. The expected frequency of depression was at 21.5% among university students (medical students) before the COVID-19 pandemic (6) at an acceptable margin of error of 5% and a design effect of 1.0. The minimum calculated sample size was 257.

### Study Measures and Procedure

The online survey (hosted on *Google Forms*) was pretested with Makerere University students, and then used for data collection from the four study sites. Students were recruited to participate using closed students’ *WhatsApp* groups and student email addresses with a weblink to the survey. Initially, approximately 100 students from each university (including student leaders) were invited to participate (convenience sampling). They were also asked to distribute the questionnaire among their peers in the four universities (snowball sampling). The online questionnaire collected information concerning socio-demographic characteristics of the participants and assessed depression using the nine-item Patient Health Questionnaire (PHQ-9).

#### Basic Information of the Participants

Data were collected including participant age (years), gender (male or female), marital status (single, cohabiting, married, and separated/divorced), religion (Christian, Muslim, none), the university they were studying (BSU, KIU, MUST, KU), and type of living residence while at the university (home, hostel, rented house, university hall or other). In addition, academic information was collected, including college or faculty, the year of study, university tuition fee sponsor [private, government, non-government organization (NGO), loan scheme, or other], and whether the student was satisfied with their most recent academic grades. Using dichotomous (yes/no) questions, participants were also asked if they had difficulty paying tuition fees, relationship problems, and history of physical and/or sexual abuse. Similarly, data concerning health factors were obtained, such as a history of mental illness or chronic medical conditions (e.g., diabetes, hypertension, asthma, HIV, etc.), and a history of substance use (cigarette/marijuana smoking, alcohol drinking).

#### Patient Health Questionnaire

Depression symptoms were assessed using the nine-item Patient Health Questionnaire (PHQ-9). Self-reported items are rated on a four-point scale ranging from 0 to 4. Each item has a discrete response such as 0 = not at all, 1 = several days, 2 = more than half the days, and 3 = nearly every day. This instrument has been internationally accepted in screening for depressive symptoms with excellent psychometric properties (30, 31). In a Ugandan setting, the PHQ-9 has also been found to have excellent psychometric properties (32–34). At a cut-off of 10, it has high specificity (85%) and high sensitivity (88%) in detecting depression based on a meta-analysis by Levis et al. (31). The PHQ-9 has been used with university students in both Uganda (6) and south-western Uganda, therefore, its questions are culturally appropriate since the instrument had good internal reliability (35, 36). In addition, the instrument has been validated for online use (37). The PHQ-9 also categorizes depression in terms of severity. More specifically, 1–4 = minimal depression, 5–9 = mild depression, 10–14 = moderate depression, 15–19 = moderately severe depression, and 20–27 = severe depression (38). A cut-off score of 10 was used to determine whether participants had depressive symptoms or not. In addition, a score of one and above on Item 9, was used to indicate the presence of suicidal ideation in the past 2 weeks. In the present study, the Cronbach alpha was 0.85 for the PHQ-9.

### Ethical Consideration

The present study was conducted in accordance with the Declaration of Helsinki 2013 and was approved by the Mbarara University of Science and Technology research ethics committee (MUSTREC #16/02-21). The Dean of Students at each of the four universities gave permission for data collection. Voluntary written informed consent was obtained from all participants.

### Statistical Analysis

STATA version 16.0 was used for data analysis. Means and standard deviations were used to summarize continuous variables that were not normally distributed, while percentages and frequencies were used to summarize categorical variables. Student’s *t*-tests and chi-square tests were performed to identify differences between depression and suicidal ideation, and independent study variables. Logistic regression analysis was used to determine the variables’ association with depression and suicidal ideation. Two separate logistic regressions were conducted to determine the factors associated with depression and suicidal ideation. Factors significant at bivariate logistic regression were tested for collinearity, and those with a variance inflation factor (VIF) of less than 3 were included in a back stepwise multivariate logistic regression model. A *p* < 0.05 for the significance level was considered at a 95% CI.

## Results

### Participants

A total of 540 students were included in the final analysis. The age of the participants ranged from 18 to 40 years, with a mean of 23.3 (*SD* ± 2.64) years. Most of the participants were male (67.22%), and the largest number of participants were from MUST (51.48%). About half of the students were satisfied with their academic performance (48.52%) and 38.15% had difficulty paying university tuition fees (Table 1).

**TABLE 1:** Distribution of the studied factors with depression and suicidal ideation (*N* = 540).

Variable	n (%)	No depression (*n* = 432, 80%)	Depression (*n* = 108, 20%)	χ^2^ (*p-*value)	No suicidal ideation (*n* = 465, 86.11%)	Suicidal ideation (*n* = 75, 13.89%)	χ^2^ (*p-*value)
Age (mean ± *SD)*	23.3 (2.64)	23.38 ± 2.75	22.98 ± 2.16	1.40 (0.162)	23.41 ± 2.74	22.61 ± 1.77	–2.43 (0.015)
**Sex**							
Female	177 (32.78)	138 (77.79)	39 (22.03)	0.68 (0.409)	149 (84.18)	28 (15.82)	0.82 (0.365)
Male	363 (67.22)	294 (80.99)	69 (19.01)		316 (87.05)	47 (12.95)	
**Current university**							
BSU	59 (10.93)	47 (79.66)	12 (20.34)	0.81 (0.847)	46 (77.97)	13 (22.03)	3.96 (0.266)
KIU	127 (23.52)	102 (80.31)	25 (19.69)		112 (88.19)	15 (11.81)	
MUST	278 (51.48)	225 (80.94)	53 (19.06)		242 (87.05)	36 (12.95)	
KU	76 (14.07)	58 (76.32)	18 (23.68)		65 (85.53)	11 (14.47)	
**Religion**							
Christian	500 (92.59)	398 (76.60)	102 (20.40)	0.76 (0.682)	430 (86.00)	70 (14.00)	0.34 (0.844)
Muslim	35 (6.48)	30 (85.71)	5 (14.29)		31 (88.57)	4 (11.43)	
None	5 (0.93)	4 (80.00)	1 (20.00)		4 (80.00)	1 (20.00)	
**Sponsor**							
Government	107 (19.81)	89 (83.18)	18 (16.82)	4.33 (0.363)	97 (90.65)	10 (9.35)	5.12 (0.275)
Loan scheme	96 (17.78)	70 (72.92)	26 (27.08)		84 (87.50)	12 (12.50)	
NGO	18 (3.33)	14 (77.78)	4 (22.22)		14 (77.78)	4 (22.22)	
Private	288 (53.33)	235 (81.60)	53 (18.40)		246 (85.42)	42 (14.58)	
Others	31 (5.74)	24 (77.42)	7 (22.58)		24 (77.43)	7 (22.58)	
**Area of residence**							
Home	35 (6.48)	30 (85.71)	5 (14.29)	3.20 (0.525)	27 (77.14)	8 (22.86)	2.77 (0.596)
Hostel	204 (37.78)	168 (82.35)	36 (17.65)		177 (86.76)	27 (13.24)	
Rentals	250 (46.30)	192 (76.80)	58 (23.20)		216 (86.40)	34 (13.60)	
University hall	45 (8.33)	37 (82.22)	8 (17.78)		40 (88.89)	5 (11.11)	
Others	6 (1.11)	5 (83.33)	1 (16.67)		5 (83.33)	1 (16.67)	
**Marital status**							
Single	497 (92.04)	396 (79.68)	101 (20.32)	0.73 (0.695)	427 (85.92)	70 (14.08)	0.55 (0.758)
Co-habiting	20 (3.70)	16 (80.00)	4 (20.00)		17 (85.00)	3 (15.00)	
Married	23 (4.26)	20 (86.98)	3 (13.04)		21 (91.30)	2 (8.70)	
**College/faculty**							
Agriculture and environment sciences	7 (1.30)	5 (71.43)	2 (28.57)	19.53 (0.012)	6 (85.71)	1 (14.29)	19.56 (0.012)
Business and management sciences	27 (5.00)	22 (81.48)	5 (18.52)		24 (88.89)	3 (11.11)	
Computing and information science	10 (1.85)	5 (50.00)	5 (50.00)		6 (60.00)	4 (40.00)	
Education and External Studies	43 (7.96)	32 (74.42)	11 (25.58)		34 (79.07)	9 (20.93)	
Engineering, designing, art, and technology	34 (6.30)	23 (67.65)	11 (32.35)		27 (79.41)	7 (20.59)	
Health sciences/Medicine	322 (59.63)	272 (84.47)	50 (15.53)		291 (90.37)	31 (9.63)	
Humanities and social sciences	6 (1.11)	3 (50.00)	3 (50.00)		4 (66.67)	2 (33.33)	
Law	11 (2.04)	10 (90.91)	1 (9.09)		10 (90.91)	1 (9.09)	
Others	80 (14.81)	60 (75.00)	20 (25.00)		63 (78.75)	17 (21.25)	
**Year of study**							
First	78 (14.44)	57 (73.08)	21 (26.92)	14.24 (0.014)	64 (82.05)	14 (17.95)	10.44 (0.064)
Second	165 (30.56)	125 (75.76)	40 (24.24)		135 (81.82)	30 (18.18)	
Third	143 (26.48)	113 (79.02)	30 (20.98)		124 (86.71)	19 (13.29)	
Fourth	96 (17.78)	82 (85.42)	14 (14.58)		86 (89.58)	10 (10.42)	
Fifth	49 (9.07)	47 (95.92)	2 (4.08)		48 (97.96)	1 (2.04)	
Sixth	9 (1.67)	8 (88.89)	1 (11.11)		8 (88.89)	1 (11.11)	
**Smoking cigarette/marijuana**							
No	529 (97.96)	424 (80.15)	105 (19.85)	0.37 (0.542)	458 (86.58)	71 (13.42)	4.74 (0.029)
Yes	11 (2.04)	8 (72.73)	3 (27.27)		7 (63.64)	4 (36.36)	
**Drinking alcohol**							
No	394 (72.96)	317 (80.46)	77 (19.54)	0.19 (0.663)	344 (87.31)	50 (12.69)	1.75 (0.186)
Yes	146 (27.04)	115 (78.77)	31 (21.23)		121 (82.88)	25 (17.12)	
**Had relationship issues**							
No	266 (49.26)	228 (85.71)	38 (14.29)	10.70 (0.001)	237 (89.01)	29 (10.90)	3.91 (0.048)
Yes	274 (50.74)	204 (74.45)	70 (25.55)		228 (83.21)	46 (16.79)	
**Had tuition constraints**							
No	334 (61.85)	279 (83.53)	55 (16.47)	6.83 (0.009)	298 (89.22)	36 (10.78)	7.08 (0.008)
Yes	206 (38.15)	153 (74.27)	53 (25.73)		167 (81.07)	39 (18.93)	
**Satisfied academic performance**							
No	278 (51.48)	205 (73.74)	73 (26.26)	14.03 (< 0.001)	224 (80.58)	54 (19.42)	14.68 (< 0.001)
Yes	262 (48.52)	227 (86.64)	35 (13.36)		241 (91.98)	21 (8.02)	
**Been sexually abused**							
No	483 (89.44)	395 (81.76)	88 (18.22)	9.07 (0.003)	421 (97.16)	62 (12.84)	4.24 (0.040)
Yes	57 (10.56)	37 (64.91)	20 (35.09)		44 (77.19)	13 (22.81)	
**Been involved in physical fighting**							
No	467 (86.48)	375 (80.30)	92 (19.70)	0.19 (0.660)	405 (86.72)	62 (13.28)	1,08 (0.298)
Yes	73 (13.52)	57 (78.08)	16 (21.92)		60 (82.19)	13 (17.81)	
**Been managed for any mental health issues**							
No	505 (93.52)	408 (80.79)	97 (19.21)	3.06 (0.080)	435 (86.14)	70 (13.86)	0.01 (0.944)
Yes	35 (6.48)	24 (68.57)	11 (31.43)		30 (85.71)	5 (14.29)	
**Had a serious medical condition**							
No	506 (93.70)	408 (80.63)	98 (19.37)	2.01 (0.156)	439 (86.76)	67 (13.24)	2.82 (0.093)
Yes	34 (6.30)	24 (70.59)	10 (29.41)		26 (76.47)	8 (23.53)	

### Prevalence of Depression and Suicidal Ideation

**FIGURE 1:**
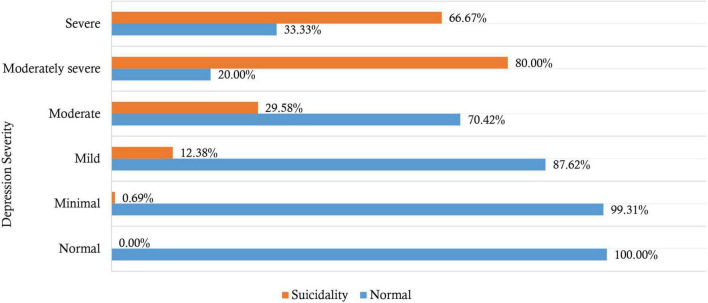
Severity of depression and suicide ideation among Ugandan University students.

Approximately 20% (95% CI: 16.71–23.63) of the students had depression based on the PHQ-9 cut-off score of 10 out of 27. The median depression symptoms score was 5 [interquartile range (IQR) = 7]. The severity of depression symptoms was: minimal (26.67%, *n* = 144/540), mild (37.41%, *n* = 202/540), moderate (13.15%, *n* = 71/540), moderately severe (4.63%, *n* = 25/540), and severe depression (2.22%, *n* = 12/540). However, 15.93% (*n* = 86/540) had no symptoms of depression (Figure 1).

A total of 75 students (13.89%) reported suicidal ideation based on a score of one and above on Item 9 of the PHQ-9. Moreover, 10.37% had thoughts of suicide for several days in the past 2 weeks (*n* = 56), 1.30% had suicidal thoughts for more than half the days in the past 2 weeks (*n* = 7), and 2.22% had thoughts of suicide nearly every day in the past 2 weeks (*n* = 12). Among individuals who scored positive for depression (at a cut-off of 10), 45.37% reported suicidal ideation (*n* = 49/108). However, 6.02% of individuals reporting suicidal ideation screened negative for depression (*n* = 26/75). There was statistically significant difference between severity of depression and having suicidal ideation (χ^2^ = 169.14, *p* < 0.001). Suicidal ideation was higher among students with moderately severe depression symptoms (80.00%), followed by severe depression (66.67%) (Figure 1).

### Relationship Between Depression and Other Variables

Depression (at a cut-off of 10) was significantly higher among students who (i) had relationship difficulties than those who did not (25.55% vs. 14.29%, χ^2^ = 10.70, *p* < 0.001); (ii) had difficulty in paying tuition fees than those who did not (25.73% vs. 16.47%, χ^2^ = 6.83, *p* = 0.009); (iii) had experienced sexual abuse than those who had not (35.09% vs. 18.22%, χ^2^ = 9.07, *p* = 0.003); and (iv) were from humanities and social sciences, and computer sciences and information sciences than those from other faculties (χ^2^ = 19.53, *p* = 0.012). Depression (at a cut-off of 10) was significantly lower among (i) fifth-year university students compared to other years of study (χ^2^ = 14.24, *p* = 0.014), and (ii) those satisfied with their current academic performance than those who were not (13.36% vs. 26.26%, χ^2^ = 14.03, *p* < 0.001) (Table 1).

### Relationship Between Suicidal Ideation and Other Variables

The mean age of individuals reporting suicidal ideation was significantly less than those without suicidality (22.61 ± 1.77 vs. 23.41 ± 2.74, *t* = -2.43, *p* = 0.015). Suicidal ideation was significantly higher among students who (i) smoked cigarettes and/or marijuana (36.36% vs. 13.42%, χ^2^ = 4.74, *p* = 0.029); (ii) had relationship difficulties (16.79% vs. 10.90%, χ^2^ = 3.91, *p* = 0.048); (iii) had difficulty in paying university tuition fees (18.93% vs. 10.78%, χ^2^ = 7.08, *p* = 0.008); and (iv) had experienced sexual abuse (22.81% vs. 12.84%, χ^2^ = 4.24, *p* = 0.040). Suicidal ideation was significantly lower among those satisfied with their current academic performance (8.02% vs. 19.42%, χ^2^ = 14.68, *p* < 0.001) (Table 1).

### Factors Associated With Depression

Table 2 shows the results of the bivariate analysis. The likelihood of depression was reduced among those (i) in the fourth and fifth year of academic study and (ii) satisfied with their current academic performance. On the other hand, the likelihood of depression was increased among those who had (i) relationship problems, (ii) difficulty paying their university tuition fees, and (iii) a history of sexual abuse. These factors were tested for collinearity, and they all had VIFs below 3, with a mean VIF of 1.04. Consequently, they were included in the final model using the backward stepwise selection method. The model had a sensitivity of 6.64%, specificity of 98.84%, a positive predictive value of 58.33%, a negative predictive value of 80.87%, and correctly classified 80.37% of depression. The goodness-of-fit *p-*value was 0.632, for the included five variables (Table 2).

**TABLE 2:** Bivariate logistic regression analysis of factors associated with depression and suicidal ideation.

Variable	Depression		Suicidal ideation	
	Crude odds ratio (95% CI)	*p-*value	Crude odds ratio (95% CI)	*p-*value
Age	0.94 (0.86–1.03)	0.163	0.86 (0.76-0.97)	0.014
**Sex**				
Female	1		1	
Male	0.83 (0.53–1.29)	0.410	0.83 (0.48-1.31)	0.366
**Current university**				
BSU	1		1	
KIU	0.96 (0.44–2.07)	0.917	0.47 (0.21-1.07)	0.074
MUST	0.92 (0.46–1.86)	0.822	0.53 (0.26-1.07)	0.076
KU	1.22 (0.53–2.78)	0.643	0.60 (0.25-1.45)	0.257
**Religion**				
Christian	1		1	
Moslem	0.65 (0.25–1.72)	0.385	0.79 (0.27-2.31)	0.671
None	0.98 (0.11–8.82)	0.982	1.53 (0.17-13.94)	0.703
**Sponsor**				
Government	1		1	
Loan scheme	1.84 (0.93–3.62)	0.079	1.39 (0.57-3.37)	0.472
NGO	1.41 (0.42–4.79)	0.579	2.77 (0.76-10.05)	0.121
Private	1.12 (0.62–2.01)	0.716	2.83 (0.98-8.20)	0.055
Others	1.44 (0.54–3.85)	0.465	1.66 (0.80-3.43)	0.175
**Area of residence**				
Home	1		1	
Hostel	1.29 (0.47–3.54)	0.627	0.51 (0.21-1.25)	0.142
Rentals	1.81 (0.67–4.88)	0.240	0.53 (0.22-1.26)	0.153
University hall	1.30 (0.38–4.38)	0.675	0.42 (0.13-1.43)	0.165
Others	1.20 (0.11–12.54)	0.879	0.67 (0.07-6.65)	0.736
**Marital status**				
Single	1		1	
Co-habiting	0.98 (0.32–3.00)	0.972	1.08 (0.31-3.77)	0.908
Married	0.59 (0.17–2.02)	0.399	0.58 (0.13-2.53)	0.470
**College/faculty**				
Agriculture and environment sciences	1		1	
Business and Management sciences	0.57 (0.08–3.82)	0.561	0.75 (0.07-8.55)	0.817
Computing and information science	2.50 (0.32–19.53)	0.382	4.00 (0.34-47.11)	0.271
Education and external studies	0.86 (0.15–5.08)	0.867	1.59 (0.17-14.93)	0.686
Engineering, designing, art, and technology	1.20 (0.20 -7.16)	0.845	1.56 (0.16-15.12)	0.703
Health sciences/Medicine	0.46 (0.09–2.43)	0.361	0.64 (0.07-5.48)	0.683
Humanities and social sciences	2.50 (0.25–24.72)	0.433	3.00 (0.20-45.24)	0.427
Law	0.25 (0.02–3.47)	0.301	0.60 (0.03-11.47)	0.734
Others	0.83 (0.15–4.64)	0.835	1.62 (0.18-14.38)	0.665
**Year of study**				
First	1		1	
Second	0.87 (0.47–1.61)	0.653	1.01 (0.50-2.05)	0.965
Third	0.72 (0.38–1.37)	0.317	0.70 (0.33-1.49)	0.354
Fourth	0.46 (0.22–0.99)	0.046	0.53 (0.22-1.27)	0.156
Fifth	0.12 (0.03–0.52)	0.005	0.09 (0.01-0.75)	0.025
Sixth	0.34 (0.04–2.88)	0.322	0.57 (0.07-4.94)	0.611
**Smoking cigarette/marijuana**				
No	1		1	
Yes	1.51 (0.39–5.81)	0.545	3.69 (1.05-12.91)	0.041
**Drinking alcohol**				
No	1		1	
Yes	1.11 (0.69–1.77)	0.663	1.42 (0.84-2.40)	0.187
**Had relationship issues**				
No	1		1	
Yes	2.06 (1.33–3.19)	< 0.001	1.65 (1.00-2.72)	0.050
**Had tuition constraints**				
No	1		1	
Yes	1.76 (1.15–2.69)	0.009	1.93 (1.18-3.16)	0.009
**Satisfied with academic performance**				
No	1		1	
Yes	0.43 (0.28–0.68)	< 0.001	0.36 (0.21-0.62)	< 0.001
**Been sexually abused**				
No	1		1	
Yes	2.43 (1.34–4.38)	0.003	2.01 (1.02-3.94)	0.043
**Been involved in physical fighting**				
No	1		1	
Yes	1.14 (0.63–2.08)	0.660	1.41 (0.73-2.73)	0.300
**Been managed for any mental health issues**				
No	1		1	
Yes	1.93 (0.91–4.07)	0.085	1.04 (0.39-2.76)	0.944
**Had serious medical condition**				
No	1		1	
Yes	1.73 (0.80–3.75)	0.161	2.02 (0.88-4.64)	0.099

In the multivariable analysis, the factors associated with depression were having relationship problems (AOR = 1.79, 95% CI = 1.13–2.81, *p* = 0.012), and having a history of sexual abuse (AOR = 2.06, 95% CI = 1.10–3.84, *p* = 0.023). Being satisfied with current academic performance (AOR = 0.50, 95% CI = 0.32–0.79, *p* = 0.003), and being in the fifth year of academic study (AOR = 0.16, 95% CI = 0.03–0.73, *p* = 0.018) reduced the likelihood of having depression (Table 3).

**TABLE 3:** Multivariate logistic regression analysis of factors associated with depression and suicidal ideation.

Variable	Depression		Suicidal ideation	
	Adjusted odds ratio (95% CI)	*p*-value	Adjusted odds ratio (95% CI)	*p*-value
Age			0.88 (0.77-1.01)	0.061
**Year of study**				
First	1		1	
Second	0.89 (0.47–1.69)	0.721	1.29 (0.61-2.74)	0.503
Third	0.78 (0.40–1.53)	0.470	1.07 (0.47-2.44)	0.872
Fourth	0.53 (0.24–1.17)	0.116	0.88 (0.33-2.33)	0.797
Fifth	0.16 (0.03–0.73)	0.018	0.20 (0.02-1.72)	0.142
Sixth	0.36 (0.04–3.34)	0.372	1.49 (0.14-15.23)	0.737
**Smoking cigarettes and/or marijuana**				
No			1	
Yes			4.83 (1.10-21.12)	0.037
**Had relationship issues**				
No	1			
Yes	1.79 (1.13–2.81)	0.012		
**Had tuition constraints**				
No	1		1	
Yes	1.34 (0.85–2.11)	0.204	1.85 (1.08-3.16)	0.024
**Satisfied with academic performance**				
No	1		1	
Yes	0.50 (0.32–0.79)	0.003	0.40 (0.23-0.70)	0.001
**Been sexually abused**				
No	1		1	
Yes	2.06 (1.10–3.84)	0.023	1.47 (0.69-3.11)	0.314

### Factors Associated With Suicidal Ideation

The likelihood of suicidal ideation was significantly lower among those (i) in the fifth year of academic study, (ii) satisfied with their current academic performance, and (iii) those who were older. On the other hand, the likelihood of suicidal ideation increased among those who had (i) difficulty paying their university tuition fees, (ii) a history of smoking cigarettes and/or marijuana, and (iii) a history of sexual abuse. These factors were tested for collinearity, and they all had VIFs below 3, with a mean VIF of 1.11. Consequently, they were included in the final model using the backward stepwise selection method. The model had a sensitivity of 4.00%, specificity of 100%, a positive predictive value of 100%, a negative predictive value of 86.59%, and correctly classified 86.67% of depression. The goodness-of-fit *p-*value was 0.934 for the included six variables (Table 2).

In the multivariable analysis, smoking cigarettes and/or marijuana (aOR = 4.83, 95% CI = 1.10–21.12, *p* = 0.037), and having financial tuition constraints (aOR = 1.85, 95% CI = 1.08–3.16, *p* = 0.024) increased the likelihood of having suicidal ideation. However, satisfaction with current academic performance reduced the likelihood of having suicidal ideation (aOR = 0.40, 95% CI = 0.23–0.70, *p* = 0.001) (Table 3).

## Discussion

In the present cross-sectional study, students from four universities in south-western Uganda during the COVID-19 pandemic were surveyed, and the prevalence of depression (at a cut-off of 10) was 20% and suicidal ideation was 13.89%. Relationship issues and a history of sexual abuse were the two factors most significantly associated with depression among students while being in the fifth year of academic study and being satisfied with current academic performance were associated with reduced depression. Smoking cigarettes and/or marijuana, and having financial tuition constraints increased the likelihood of reporting suicidal ideation. However, similar to depression, satisfaction with the current academic performance reduced the likelihood of reporting suicidal ideation.

During the COVID-19 pandemic, higher mental health problems have been reported among students. For instance, the prevalence of depression ranged from 46.92% to 82.4% among Bangladeshi students, as reported in a systematic review (13). Moreover, a recent meta-analysis reported a pooled prevalence of 37% depression among 436,799 students in studies published before September 20, 2020 (12). However, the depression prevalence of the present study (20%) was markedly lower than the previously reported prevalence rate of 80.7% during the first wave of the pandemic (June to July 2020) in a Ugandan study among 321 university students using the 21-item Depression, Anxiety and Stress Scale (4). The difference may be because the present study used a different instrument to screen for depression, with different sensitivities and specificities for assessing depression. In addition, Najjuka et al.’s (4) study involved all university students during the complete institutional lockdown in the first wave of the pandemic, when the levels of uncertainty about the disease were higher and there was associated higher levels of depression among students as reported by other studies (14).

The prevalence of depression in the present study was higher than that reported among non-medical undergraduate university students prior to the introduction of the peer counseling services at Makerere university, 2000–2001 at 16.2%; and medical undergraduate university students following peer counseling introduction (2002) at 4.0% (7). Despite the present study being conducted during a period when peer counseling and other psychological intervention for student mental ill-health is well established among all Ugandan universities, the prevalence of depression reported by Ovuga et al. (7) may have been lower because the study was conducted before the COVID-19 pandemic—a period that significantly increased negative psychological effects among many students (11). However, the depression prevalence of 20% in the present study is slightly lower than 21.5% reported in a study done the year before the pandemic (May–July 2019) among 331 Makerere University medical students using the same instrument (6). The similarity between the study findings and Olum et al. (6), coupled with the significant difference from the only study assessing depression among university students during the pandemic in Uganda (4), could possibly indicate that students have adjusted to the COVID-19 situation and associated stressors, the so-called “new normal,” as reported by other researchers (39). Therefore, depression may be associated with other stressors rather than being pandemic-related.

For instance, in the present study, depression among students was associated with having a history of sexual abuse, a finding similar to other studies among university students. It is also known that individuals who experience sexual abuse during childhood often experience associated depression in adulthood (40). In addition, the severity of depression can be accelerated by the abusive behaviors of the current sexual partner (17, 41). Since students in an abusive relationship have fewer protective factors against depression associated with a supportive romantic relationship, such as comfort, emotional support, and companionship (42). In addition, relationship problems cause significant psychological distress due to disappointment and unmet expectations (43).

Satisfaction with academic grades appeared to be a protective factor against depression and suicidal ideation. Good academic performance is associated with being successful in life (44); therefore, an individual feels positive about their future when their performance is good, and this may help protect them from depression. Conversely, poor academic performance has consistently been associated with depression (45). In Uganda, education is considered the “key to success” (46). Students are expected to meet extremely high expectations from their parents and society to succeed (47). Therefore, it is perhaps unsurprising that students with financial tuition constraints had an increased likelihood of reporting suicidal ideation because if they do not have the ability to fully pay for their university fees, they may lose hope for the future and experience suicidal ideation. However, it has also been reported that some students use their tuition fees to gamble, therefore, they have financial problems if they lose, leading to suicidal behaviors and suicide (48). In Uganda, law and medicine degree programs are pursued for a minimum of 5 years, and culturally these courses are associated with a successful future. Therefore, studying in the fifth-year means being near the end of the undergraduate studies for students, and the dream of future success in life is also nearing. Other researchers studying Ugandan university students have also reported the protective nature of being in the final year against depression during the COVID-19 pandemic (4). Also, by the time a student reaches the fifth year of study, they have been more likely to be exposed to all the conditions around campus and developed coping mechanisms against depression. For example, some have already coped with experiencing romantic breakups, poverty while on campus, and have gotten a peer support system through the friends they acquired over the years.

Just under half of the participants who screened positive for depression reported suicidal ideation in the past 2 weeks (45.37%). This was similar to 48.4% of individuals with major depressive disorder at a mental health facility in Ethiopia (49). The prevalence of suicidal ideation in the present study (13.89%) was lower than 22.7% from a study conducted among university students in another African country, Libya, during the COVID-19 pandemic in the months of April and May 2020 based on Item 9 of the PHQ-9 (50). This difference in prevalence rates may be due to the following reasons: (1) during the time of data collection, Libya was still experiencing a civil war which comes with multiple mental challenges, including thoughts of suicide, (ii) the study was done at a period when individuals worldwide were having higher levels of stress, anxiety, suicidal behaviors and other mental challenges (i.e., early stages of the pandemic) (51); and (iii) the sample only involved medical students who have been reported to experience higher suicidal behaviors compared to other students (52–54).

The prevalence of suicidal ideation in the past 2 weeks in the present study was also lower than 18.04% in a US study involving college students during the month of May 2020 (55). However, the prevalence was higher than 12.9 and 7.3% from studies among university students in China during February and March 2020, respectively (56, 57). Despite the studies in China being conducted earlier, the students were exposed to constant sources of stress during the pandemic, and they would have developed coping strategies to overcome stress complications such as depression and related suicidal behaviors. Smoking cigarettes and/or marijuana was associated with suicidal ideation, a finding consistent with other studies prior to the COVID-19 pandemic (58). Substance use increased during the COVID-19 pandemic and led to multiple mental challenges, including suicide (59–63). Many individuals use addictive substances to cope with stress, and marijuana, in particular, was believed by some Ugandans to treat COVID-19 symptoms (61). Some of the individuals trying to cope with stress by using psychoactive substances could have had suicidal ideation not secondary to the smoking of substances.

## Limitations

The present study has a number of limitations. First, it was cross-sectional; therefore, causal relationships between the variables cannot be determined. Second, the study was conducted during the COVID-19 pandemic, which could also independently cause depression among some university students due to uncertainty about their academic progress, source of income, their jobs (for the employed participants), and worries about life. Third, another issue was that anyone who was given access to the survey web-link could respond to it; therefore, there is no guarantee that all responses were from university students only. Fourth, the study design relied on the participants’ memory, which is subject to recall bias. Fifth, this study did not access all the potential variables that could have led to an increase in depression among university students such as disruption of face-to-face teaching classes. Future research should explore these factors to better understand the factors related to depression and the pandemic to reduce such mental consequences in future pandemics. Finally, the study design did not allow for the determination of a true response rate since *WhatsApp* and emails are unable to track the number of individuals who viewed the link, especially when shared through groups.

## Conclusion

The present study found a lower prevalence of depression among university students during the COVID-19 pandemic, similar to pre-pandemic prevalence among university students, and this was associated with having had relationship problems and a history of sexual abuse. However, being satisfied with current academic performance and studying in the fifth year appeared to be protective factors. Therefore, universities in Uganda should implement measures to provide psychological support for students with problems concerning tuition fee challenges, relationships, and past sexual abuse. These can include peer support groups and routine talks about dating and relationships to prepare the students for any outcome. In addition, finalists should be encouraged to mentor other students and teach them strategies to overcome some of the psychological stressors experienced while at university. Also, all students with depression should be screened for suicidal behaviors.

## Data Availability Statement

The raw data supporting the conclusions of this article will be made available by the authors upon reasonable request.

## Ethics Statement

The studies involving human participants were reviewed and approved by the Mbarara University of Science and Technology Research Ethics Committee. The patients/participants provided their written informed consent to participate in this study.

## Author Contributions

IA, MMK, EN, MM, JK, MK, NA, IM, NW, and GR: conception and design of the study. IA, MMK, EN, MM, JK, MK, NA, RN, IM, and NW: data collection and its coordination. GR, MAM, MG, and FB: supervision. MMK and IA: formal analysis and data cleaning. IA: initial draft. IA, MMK, EN, MM, JK, RN, MK, NA, IM, SN, NW, FB, MAM, MG, and GR: review of the manuscript. MG: final editing. All authors approved the final version of the manuscript.

## Conflict of Interest

The authors declare that the research was conducted in the absence of any commercial or financial relationships that could be construed as a potential conflict of interest.

## Publisher’s Note

All claims expressed in this article are solely those of the authors and do not necessarily represent those of their affiliated organizations, or those of the publisher, the editors and the reviewers. Any product that may be evaluated in this article, or claim that may be made by its manufacturer, is not guaranteed or endorsed by the publisher.

## Abbreviations

BSU: Bishop Stuart University
KIU: Kampala International University Western campus
MUST: Mbarara University of Science and Technology
KU: Kabale University
PHQ-9: Patient Health Questionnaire 9.
